# Prokineticin receptor-1 signaling promotes Epicardial to Mesenchymal Transition during heart development

**DOI:** 10.1038/srep25541

**Published:** 2016-05-06

**Authors:** Himanshu Arora, Mounia Boulberdaa, Rehana Qureshi, Verda Bitirim, Adeline Gasser, Nadia Messaddeq, Pascal Dolle, Canan G. Nebigil

**Affiliations:** 1 CNRS, Université de Strasbourg, UMR7242, Ecole Supérieure de Biotechnologie de Strasbourg, Illkirch, France; 2 Institut de Génétique et de Biologie Moléculaire et Cellulaire (IGBMC), CNRS, UMR 7104 and INSERM Unité 964, Université de Strasbourg, Illkirch-Strasbourg, France

## Abstract

The epicardium plays an essential role in coronary artery formation and myocardial development. However, signals controlling the developing epicardium and epicardial-mesenchymal transition (EMT) in the normal and diseased adult heart are studied less rigorously. Here we investigated the role of angiogenic hormone, prokineticin-2 and its receptor PKR1 in the epicardium of developing and adult heart. Genetic ablation of PKR1 in epicardium leads to partial embryonic and postnatal lethality with abnormal heart development. Cardiac developmental defects are manifested in the adult stage as ischemic cardiomyopathy with systolic dysfunction. We discovered that PKR1 regulates epicardial-mesenchymal transition (EMT) for epicardial-derived progenitor cell (EPDC), formation. This event affects at least three consequential steps during heart development: (i) EPDC and cardiomyocyte proliferation involved in thickening of an outer compact ventricular chamber wall, (ii) rhythmicity, (iii) formation of coronary circulation. In isolated embryonic EPDCs, overexpression or activation of PKR1 alters cell morphology and EMT markers via activating Akt signaling. Lack of PKR1 signal in epicardium leads to defective heart development and underlies the origin of congenital heart disease in adult mice. Our mice provide genetic models for congenital dysfunction of the heart and should facilitate studies of both pathogenesis and therapy of cardiac disorders in humans.

Congenital heart disease is the most common human malformation. Alterations in cardiac development may lead to a variety of congenital diseases. The epicardium layer of epithelial cells covering the myocardial surface has emerged as physiologically relevant tissue in cardiac development. One of the important developmental mechanism in the heart is epicardial-to-mesenchymal transformation (EMT)^1^. Epicardium undergoes EMT to form epicardial-derived progenitor cells (EPDCs) that contribute to the development and maturation of many cardiac cell types^2^. Therefore, identification of the factor that regulates of EMT in epicardium is essential to understand the etiology of heart diseases.

Prokineticin-1 and 2 are potent angiogenic factors that use two G-protein coupled receptors (GPCRs); PKR1 and PKR2^3^. Prokineticin-2 is the most potent agonist of these two receptors. Expression of prokineticin-2 and PKR1 are increased in a mouse model of acute myocardial infarction^4^ and decreased in hearts of end state heart failure patients^5^. These observations implicate a central role for prokineticin-2/PKR1 signaling in myocardial development and coronary repair^6^. Prokineticin-2 via PKR1 induces coronary endothelial cell proliferation, migration, and angiogenesis *in vitro and in vivo*^7^. Activation or overexpression of PKR1 protects cardiomyocytes against hypoxic insult^5^. However, PKR2 is involved in endothelial cell fenestration and development of hypertrophy in the heart^8^. Transient PKR1 gene transfer after coronary ligation in the mouse model of myocardial infarction reduces mortality and preserves heart function by promoting cardiac angiogenesis and cardiomyocyte survival^5^. Transgenic mice specifically overexpressing PKR1 in cardiomyocytes (utilizing the α -MHC promoter) display increased neovascularization^9^.

Myocardial PKR1 signaling was shown to regulate epicardium-derived cell (EPDC) differentiation in a paracrine manner to induce neovascularization in mouse heart^9^. Cardiomyocytes overexpression of PKR1 up regulates prokineticin-2, its own ligand. Prokineticin-2 then acts as a paracrine factor, promoting heterogeneous EPDC differentiation into endothelial and smooth muscle cells (SMC). These prokineticin-2 effects were abolished in EPDC derived from PKR1-null mutant hearts, demonstrating PKR1 involvement^9^. In addition, prokineticin/PKR1 signaling reprograms adult EPDCs in a cell autonomous way^9^. Global loss of PKR1 impairs heart morphogenesis, leading to cardiomyopathy and cardiac dysfunction^10^. However, the cellular and molecular mechanisms underlying these pathologies have not been reported yet^11^.

In this study we investigated the effect of *PKR1* deficiency in epicardial cells on heart function and development, focusing on the formation of EPDCs by epicardial EMT. We used the Gata5 (G5)-Cre^12^ and Wt1GFP cre transgenic lines^13^ and generate PKR1 mutant lines. Since Wt1 is expressed in epicardium^14^, Wt1GFP cre mice provide additional advantages to trace these progenitor cells in the heart. We showed that loss of PKR1 in epicardium impaired EMT events, affecting proliferation and survival of these cardiac progenitor cells, leading to developmental and functional defects in the heart. Our results designate PKR1 signaling as another angiogenic system involved in epicardial development via Akt activation in modulating EMT.

## Results

### Verification of loss of PKR1 in heart of mutant lines

**Figure 1:**
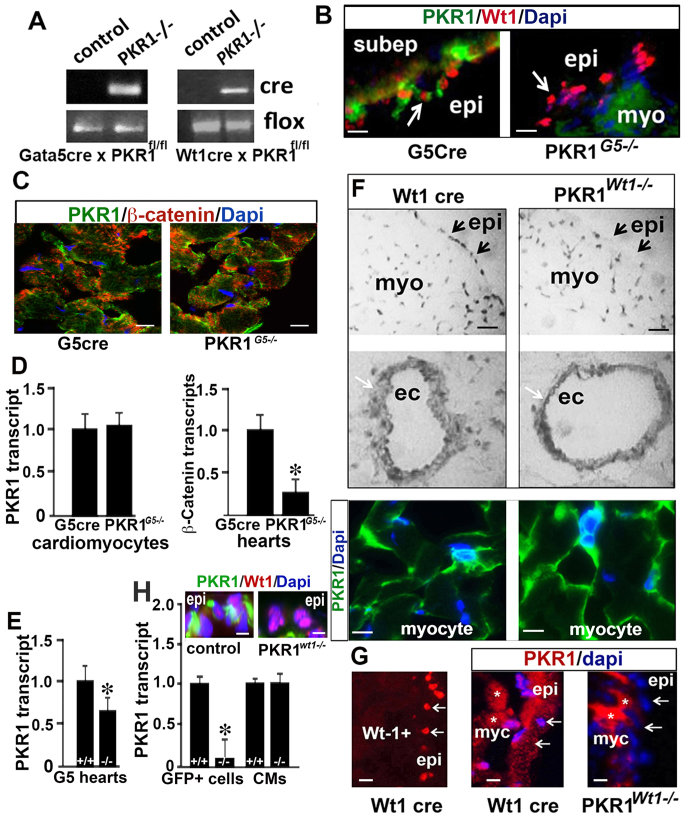
Mutant mice exhibit loss of PKR1 in the epicardium. (**A**) Representative genotype analysis of PKR1^*Wt1−/−*^ and PKR1G5^*−/−*^ mice. Genomic DNA was amplified with oligonucleotide primers detecting *Cre* and PKR1lox/lox alleles. PKR1^*Wt1−/−*^ mice and PKR1^*G5−/−*^ mice harbor the *Cre* transgene and are homozygous for the PKR1 floxed allele (PKR1lox/lox). Control mice are *Cre* negative and they are PKR1lox/+ (**B**) Double immunostaining for PKR1 and Wt1 on cryosectioned heart from control (left side) and PKR1^*G5−/−*^ (right side) mice, showing the loss of PKR1 in epicardium. (**C**) Illustration shows identical expression of PKR1 in PKR1^*G5−/−*^ myocytes as compare to control group. PKR1^*G5−/−*^ cardiomyocytes did not show any changes in PKR1 transcripts. PKR1^*G5−/−*^ hearts had a significantly lower transcript of β -catenin. (**E**) PKR1^*G5−/−*^ neonatal mice express lower levels of PKR1 mRNA in the hearts as detected by qPCR. *p <  0.05, n =  3, (at least 5 mice per genotype). (**F**) PKR1-immunohistochemistry, utilizing peroxidase kit (E15.5dpc) illustrates a loss of PKR1 in the epicardium (upper), despite of no modification of PKR1 expression in the endothelium (middle) and myocardium of PKR1^*Wt1−/−*^ hearts (lower), n =  4. (**G**) Immune fluorescent (E10.5 dpc) staining for Wt1 (red) shows specific staining for epicardium. In the same heart section PKR1 (red) was not expressed in the epicardium. (**H**) Representative illustration shows loss of PKR1 protein in Wt1^+^ epicardium of PKR1^*Wt1−/−*^ hearts. mRNA was severely reduced in GFP-positive (Wt1^+^) epicardial cells, as observed on FACS-sorted GFP^+^ cells from P14.5 or 16.5 mice hearts. However, PKR1 levels are unaffected in myocardial cells. Data are expressed as mean ±  SEM; *p <  0.05; n =  4. epi: epicardium, myo: myocardium, ec:endothelial cells, fl: floxed.

Loss of PKR1 in epicardium was achieved by interbreeding Gata5-Cre (PilarRuiz-Lozano, Stanford University, Stanford, CA, USA) and Wt1-CreGFP (The Jackson Laboratory, Bar Harbor, ME, USA) transgenic mice with mice harboring a floxed PKR1 allele (Gata5cre X PKR1^fl/fl^ or Wt1cre X PKR1^fl/fl^). Examples of genotyping analyses are shown in Fig. 1A, and primers sequences are given in Table S1 (Supplementary Material). PKR1 protein (green) was absent from Wt1-positive epicardial cells, but it was present in myocardium of newborn PKR1^*G5−/−*^ hearts with diminished subepicardium (Fig. 1B). Accordingly, PKR1 levels were identical in the myocardium (Fig. 1C) and in isolated cardiomyocytes of PKR1^*G5−/−*^ mice (Fig. 1D left histogram). Low level of β -catenin expression in the PKR1^*G5−/−*^ neonatal hearts may be associated with defective EMT during embryogenesis (Fig. 1D right histogram). Immunohistochemistry revealed severe loss of PKR1 in the epicardium (Fig. 1F upper) despite of no modification of PKR1 expression in the endothelium (Fig. 1F middle) and myocardium (Fig. 1F lower) of PKR1^*Wt1−/−*^ hearts at E15.5 dpc. At E10.5 dpc, loss of epicardial PKR1 was evident only in the PKR1^*Wt1−/−*^ hearts (Fig. 1G). PKR1 protein (green) was also absent in the Wt1^+^ (nuclear-red) epicardium of PKR1^*Wt1−/−*^ hearts (Fig. 1H upper illustration). Wt1^GFP+^ cells are lacked PKR1 transcript (Fig. 1H left), however normal PKR1 expression was found in the cardiomyocytes of the PKR1^*Wt1−/−*^ hearts (Fig. 1H right). Although the epicardial-restricted *Gata5*^*Cre*^ and *Wt1*^*GFP/Cre*^ mice displayed ectopic Cre expression in the endocardial/endothelial and myocardial cells of the developing heart^15^, our data demonstrate that both G5-Cre and Wt1-CreGFP efficiently removes PKR1 from the epicardium without significantly altering myocardial or endothelial PKR1 expression.

### Impaired heart functions in adult PKR1^G5*−/−*^ mice

**Figure 2:**
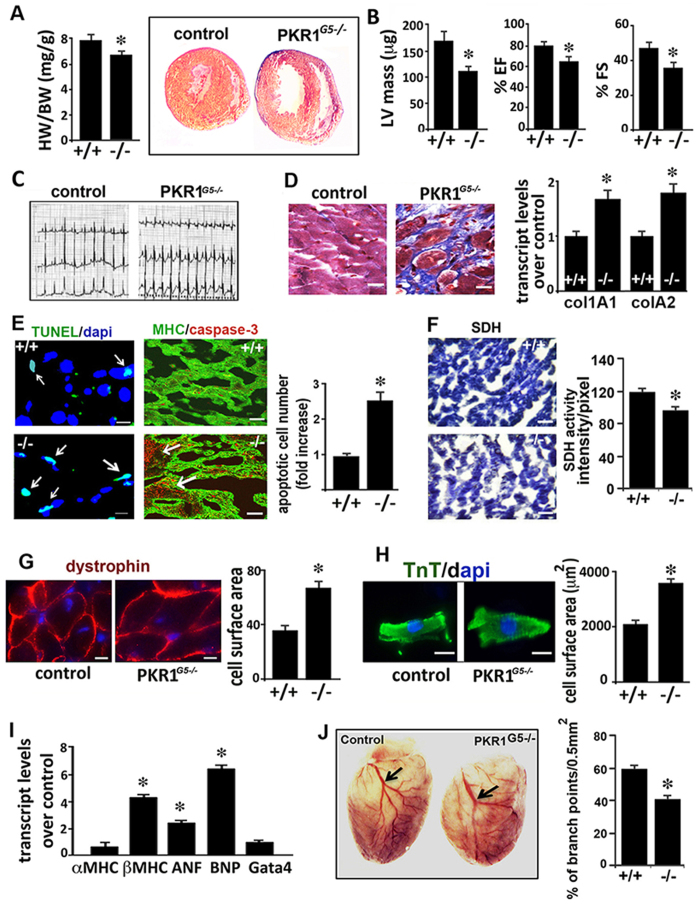
Adult PKR1^*G5−/−*^ mice developed ischemic cardiomyopathy. Histogram shows a low heart weight (mg) to body weight (g) ratio (n =  5) in PKR1^*G5−/−*^ mice. Histological analysis (Mallory tetrachrome staining) of control and PKR1^*G5−/−*^ adult heart sections show a thinner ventricular wall in PKR1^*G5−/−*^ hearts. (**B**) Echocardiographic analyses showed left ventricular mass, ejection fraction (EF) and fractional shortening (FS) to be lower in PKR1^*G5−/−*^ hearts (n =  6). (**C**) ECG revealed ST segment depression, development of Q waves and negative T-waves in mutant hearts. (**D**) Mallory staining and qPCR analyses revealed fibrosis (blue) and increased levels of collagen transcripts in PKR1^*G5−/−*^ hearts. Data are expressed as mean ±  SEM; *p <  0.05; n =  3, 4 mice per genotype). (**E**) Illustrations and quantification of TUNEL positive nuclei in adult cardiac samples shows an increase in apoptosis in PKR1^*G5−/−*^ hearts. (**F**) Succinate dehydrogenase (SDH) staining reveals low level of SDH activity as an indicator of mitochondrial abnormalities. (**G**) Dystrophin stained hearts show increased cardiomyocytes surface in the PKR1^*G5−/−*^ hearts. (**H**) Isolated PKR1^*G5−/−*^ cardiomyocytes display increased surface area (histogram, *n* =  95, two hearts). (**I**) qPCR analysis of genes involved in hypertrophic response (α -, β -MHC, ANF, BNP, Gata 4) in PKR1^*G5−/−*^ hearts Data are expressed as mean ±  SEM. *p <  0.05 versus respective control, n =  3, 6 mice per genotype). (**J**) Tail Evans blue intravenous injection reveled reduced number of branching points, indicating impaired coronary vessel growth in mutant hearts (a least 5–6 mice per genotype). For immunohistochemical, TUNEL, SDH and surface analyses, 10 high-power microscopic fields (× 40) from 5–6 sections per hearts (4–5 mice per genotype) were analyzed. Data are expressed as mean ±  SEM. *p <  0.05 versus respective control.

Although PKR1^*G5−/−*^ mice were born at the expected Mendelian ratio, 11 ±  2% of these mice developed heart failure and postnatal premature lethality. Histological analyses showed that PKR1^*G5−/−*^ mutants (24 weeks old) had thinner ventricular wall (Fig. 2A right), also reflected in a low heart weight to body weight ratio (Fig. 2A, histogram). Echocardiographic analyses revealed low ventricular mass and impaired ejection and shortening fractions in adult PKR1^*G5−/−*^ mice, demonstrating systolic and diastolic dysfunctions (Fig. 2B, Table 2 in Supplementary Materials). Interestingly, isovolumetric contraction time (IVCT), defined as the interval between mitral valve closure and aortic valve opening, was prolonged, indicating an impairment of the initial left ventricular contractile phase (Table 2, Supplementary Materials). ECG analysis revealed signs of ischemia with an ST-segment depression and negative T-waves in the mutants (Fig. 2C). Severe interstitial fibrosis (blue) detected by Mallory’s tetrachrome staining on cryosectioned heart samples with increased collagen transcript levels was evident in adult mutant hearts (Fig. 2D).

Ventricular hypoplasia and impaired function can be due to a marked caspase-3^+^/MHC^+^ cardiomyocyte loss due to increased apoptosis detected by Tunel analyses in mutant hearts (20 ±  2 Tunel^+^ nuclei/10^4^ myocytes) compared to control hearts (8 ±  1 Tunel^+^ nuclei/10^4^ myocytes) (Fig. 2E). A low level of succinate dehydrogenase (SDH) activity in the subepicardial area in PKR1^*G5−/−*^ hearts indicates low oxidative capacity of the mitochondria (Fig. 2F). Morphometric analysis of the dystrophin-stained heart tissues (Fig. 2G), and isolated cardiomyocytes, showed a significant increase in cell size in PKR1^*G5−/−*^ mice compared with controls (Fig. 2H). In addition, significant increases in markers of myocardial hypertrophy such as *β-myosin heavy chain (MHC), atrial natriuretic factor* (ANF), and *brain natriuretic peptide* (BNP) were observed (Fig. 2I). This data indicate that the viable cardiomyocytes in mutant hearts developed hypertrophy to compensate for functional defects. Thus, mutant mice developed ischemic cardiomyopathy with systolic dysfunction.

A less developed coronary network was evident in the adult PKR1^*G5−/−*^ heart with reduced number of branching points visualized by tail intravenous Evans Blue (%0.5) injection (Fig. 2J). In concert with these findings, PECAM-1 positive capillary number and α -SMA positive coronary artery numbers were reduced (Supplemental Material Figure S1A). Survival rates of the mutant mice were dramatically reduced after coronary ligation, a mouse model of myocardial infarction (Supplemental Material Figure S1B).

Cardiomyopathy seems to have resulted from a default in vasculogenesis and excessive cardiomyocyte death. These data indicate that the lethality among the adult mutant mice is likely due to impaired heart functions.

### Hypoplastic heart with vascular defects in PKR1^G5*−/−*^ neonates

**Figure 3:**
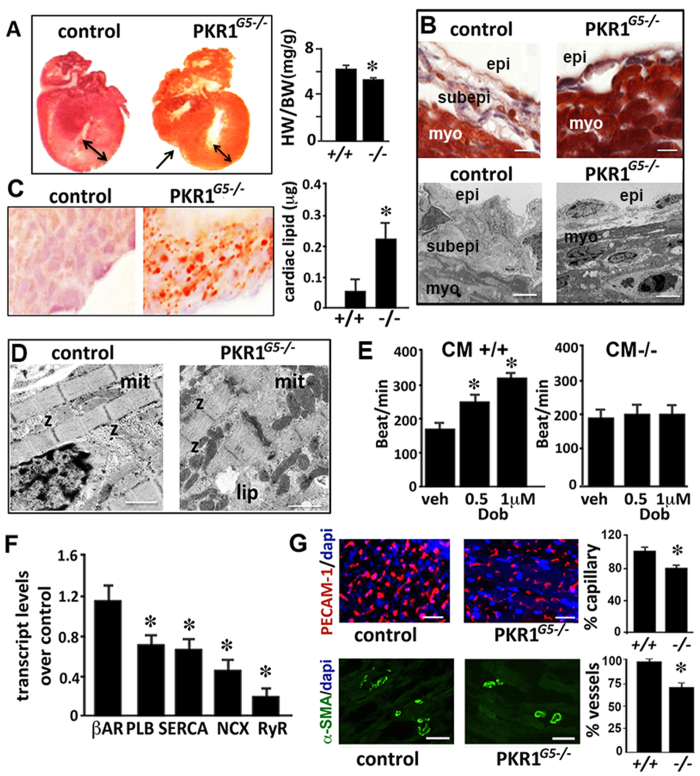
PKR1^*G5−/−*^ neonatal hearts developed hypoplasia with defects in vascularization and functions. (**A**) Histological analysis (Mallory tetrachrome staining) of control and PKR1^*G5−/−*^ neonatal heart sections, showing a thinner ventricular wall in PKR1^*G5−/−*^ hearts. Histogram shows the ratio of heart (g) to body weight (g) (n =  6). (**B**) Histological analyses and electron microscopy (lower panels) demonstrate reduced subepicardial area and thinner epicardium in PKR1^*G5−/−*^ hearts. (**C**) Oil red O staining showing lipid accumulation in the neonatal PKR1^*G5−/−*^ hearts. Extracted cardiac lipid levels were increased in PKR1^*G5−/−*^ hearts (6 mice per genotype). (**D**) Electron microscopic analyses show lipid accumulation and increased number of mitochondria. (**E**) The mutant cardiomyocytes were unable to respond to dobutamine (0.5–1 μ M) (3 different cardiomyocyte cultures, 12 mice per genotype). (**F**) qPCR analysis revealed reduced transcript levels of calcium handling genes (β -AR, PLB, SERCA, NXC, and RyR) as indicators of impaired contractility in PKR1^*G5−/−*^ hearts (n =  4, 4 mice per genotype). (**G**) Illustrations and histograms show regression of the capillary network detected by PECAM-1 (upper panels) and vascular network detected by α -SMA staining (lower panels) in mutant hearts. For Oil-red-O, PECAM-1 and α -SMA analyses, 10 high-power microscopic fields (×40) from 5–6 sections per heart were analyzed (4–5 mice per genotype). Data are expressed as mean ±  SEM. *p <  0.05 versus respective control.

Mutant neonates displayed a pronounced reduction in cardiac size and weight (Fig. 3A). The subepicardial space was diminished in PKR1^*G5−/−*^ mice, as shown by Mallory staining of the cryosectioned hearts (Fig. 3B upper panel) and EM studies (Fig. 3B lower panel). Oil red O staining showed numerous small droplets of neutral lipids throughout the cytosol of PKR1^*G5−/−*^ cardiomyocytes (Fig. 3C). In concert with this finding, extracted total lipid levels in mutant hearts were elevated (Fig. 3C Histogram). Electron microscopic analyses demonstrated preserved cardiac cytoarchitecture, yet an abnormally high presence of cytosolic lipid droplets and mitochondria (Fig. 3D). Abnormal mitochondria and lipid accumulation in PKR1^*G5−/−*^ cardiomyocytes might account for the lower beating rate in response to dobutamine (Fig. 3E). Accompanying reductions in transcript levels of calcium handling genes (phospholamban, SERCA Na^+^/Ca^++^ exchanger and ryanodine receptor) reflected impaired cardiac contractility found in the mutants (Fig. 3F). The neonatal PKR1^*G5−/−*^ mice had a less developed capillary network within the subepicardial layer of the dorsal wall of the ventricles, as shown by endothelial cell-specific PECAM-1 staining (Fig. 3G upper panels). α -SMA immunostaining demonstrated lower number of vessels in mutant hearts (Fig. 3G, lower panels). These data indicate that these mutant mice have congenital heart diseases.

### PKR1^Wt1*−/−*^ embryos have embryonic lethality due to impaired proliferation and survival of GFP^+^ cells

**Figure 4:**
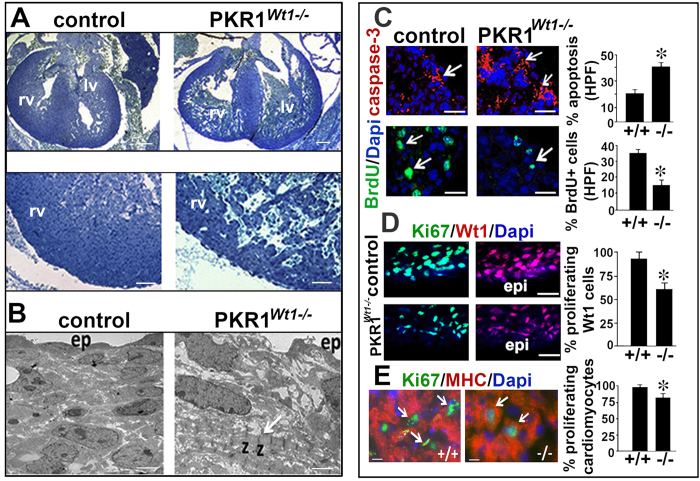
Cardiac growth defects in PKR1^*Wt1−/−*^ embryos. (**A**) Toluidine blue staining of 14.5 dpc wild type (left) and PKR1^*Wt1−/−*^ (right) heart sections revealed a severe myocardial hypoplasia in mutant hearts. (**B**) Electron microscopic (EM) analyses revealed immature sarcomeric Z bands in the subepicardium of mutant hearts. ra: right atrium, rv: right ventricle, la: left atrium, lv: left ventricle, ep: epicardium. (**C**) Representative illustration and quantification of hearts stained with cleaved caspase 3 (active caspase 3, upper panel) BrdU (lower panel). An increase in apoptosis and hypoproliferation in the PKR1^*Wt1−/−*^ hearts was evident. Arrows show caspase-3^+^/Dapi^+^ apoptotic and BrdU^+^/Dapi^+^ proliferating cells. (**D**) Wt1^+^/Ki67^+^ proliferating epicardial cell numbers (upper panel) and (**E**) MHC^+^/Ki67^+^ proliferating cardiomyocyte (CM) numbers were reduced in the PKR1^*Wt1−/−*^ hearts. For caspase-3, BrDU, Wt1 and MHC analyses, 10 high-power microscopic fields (×40) from 5–6 sections per organ were analyzed (5–6 mice per genotype). Data are expressed as mean ±  SEM. *p <  0.05 versus respective control.

Next we generated a second epicardial specific PKR1 inactivation in mice (W PKR1^*Wt1−/−*^) by using Wt1-GFPCre transgenic mice to gain insight to the cellular and molecular mechanism of the phenotype. We found that PKR1^*Wt1−/−*^ mice suffer from a 15 ±  2% lethality (*P* <  0.05, *n* =  90) at fetal stages (between 12.5 and 16.5 dpc). Histological analysis of hearts from PKR1^*Wt1−/−*^ embryos (14.5 dpc) revealed a bifid apex, cardiac compact zone hypoplasia with reduced ventricular expansion, and septal defects. Dilation of the left atrium was also apparent (Fig. 4A, upper and middle panels). At 14.5 dpc, in wild type embryos the vast majority of myocytes adjacent to the epicardium remained relatively undifferentiated. Hence subepicardial cardiomyocytes rarely contained essential components of contractile myocytes, such as sarcomeres, (the contractile unit found in differentiated myocardial cells), intercalated disks or sarcoplasmic reticulum in the control hearts (Fig. 4B). In PKR1^*Wt1−/−*^ mutants, about 50% of these subepicardial cells exhibited premature differentiation, hence contained striated myofibrils, displaying on average 10 consecutive sarcomeres with “z” bands. Increased active-caspase-3 staining (GFP^+^) in the cytoplasm (showing increased apoptosis) was accompanied by a reduction of BrdU-positive proliferating cells in the PKR1^*Wt1−/−*^ hearts (Fig. 4C). Interestingly, in control embryos the proliferating Wt1^+^/Ki67^+^ cells were found not only in a subset of epicardial but also in subepicardial mesenchyme (Fig. 4D). This expansion of subepicardial mesenchyme is suggestive of normal EMT process^16^. In mutant hearts, although the number of cells was decreased, a few subepicardial cells stained with both Wt1 and Ki67 (Figure S2A), indicating impaired EMT in mutant epicardium. These data clearly show that loss of PKR1 in epicardium effectively impairs proliferation and survival in epicardial progenitor cells. In concert with these *in vivo* findings, prokineticin-2 has also promoted proliferation and survival in isolated EPDCs that was blocked by PI3K/Akt inhibitor LY 40092 (Figure S2B–D).

In PKR1^*Wt1−/−*^ hearts, numbers of MHC^+^/Ki67^+^ cardiomyocytes were also reduced (Fig. 4E), indicating that epicardial-PKR1 signaling is important for cardiomyocyte proliferation during embryogenesis. Next, we compared the proliferation rate of cardiomyocytes (wild type) when they cultured in the conditioned media of epicardial cells derived from control or mutant hearts. Cardiomyocyte growing rate was significantly lower in the conditioned media of the mutant EPDCs, showing lack of epicardial-derived proliferative signaling in mutant hearts (Supplemental Material Figure S3A). All together these data clearly show that cardiomyocyte phenotype is resulted from lack of epicardial paracrine signal due to loss of PKR1 in epicardium.

Consequently, numbers of PECAM-1 positive capillaries (Supplemental Material Figure S3B) and α -SMA positive coronary arteries (Figure S3C) were reduced in PKR1^*Wt1−/−*^ hearts. Abnormal coronary artery development in PKR1^*Wt1−/−*^ hearts was also demonstrated with Evans blue staining (Figure S3D).

These data clearly show that loss of PKR1 in the epicardium results in impaired proliferation and survival of EPDCs cells, affecting cardiomyocyte proliferation and vascular formations.

### PKR1^Wt1*−/−*^ embryos have deregulated EMT in the heart

**Figure 5:**
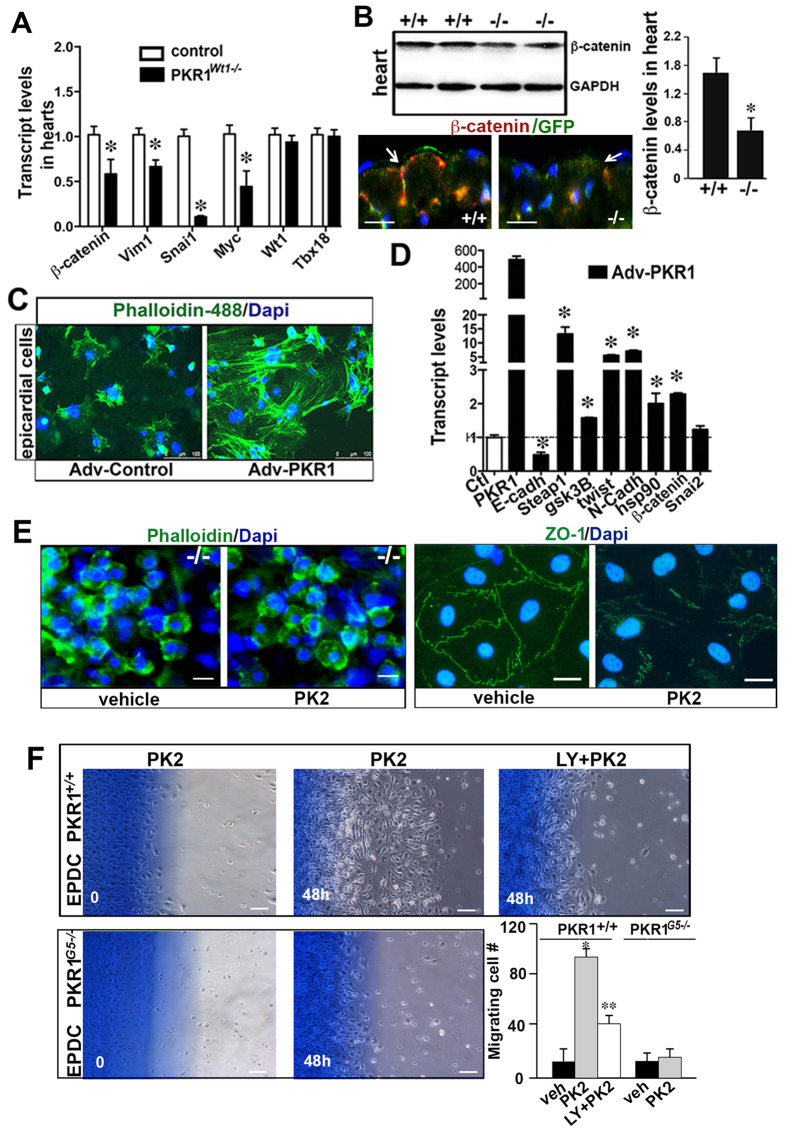
Deregulated EMT in epicardium of PKR1^*Wt1−/−*^. (**A**) EMT markers (*β-catenin*, SNAI1 *vim1 and myc)* but not epicardial markers *(Tbx18 and Wt1)* were reduced in mutant hearts (n =  3, 5–6 individual hearts). (**B**) Representative western Blot analyses on whole heart extracts of mutant and control mice, utilizing β -catenin antibody and GAPDH as an internal control. Illustration shows β -catenin expression profile in the epicardium. Histogram shows that β -catenin protein levels were decreased in PKR1^*Wt1−/−*^ hearts (at least 3 times repeated, 5–6 mice per genotype, *p <  0.05). (**C**) PKR1 overexpression of epicardial cells significantly increased F-actin organization that was visualized by Phalloidin staining, indicating a mesenchymal-like cell formation. (**D**) Overexpression of PKR1 in embryonic epicardial cells by Adv-PKR1 increased EMT marker levels (n =  3). Data are expressed as mean ±  SEM (*p <  0.05). (**E**) Phalloidin staining in PKR1 deficient cells shows octagonal cell shapes without actin fibers after PK2 treatment. Representative illustration of ZO-1 internalization in epicardial cells 1h after PK2 treatment. (**F**) Explant cultures derived from control or PKR1^*G5−/−*^ embryonic hearts exposed to wound and healing process in the presence of PK2. PI3K/Akt inhibitor LY294002 reduced PK2-mediated migration. Quantification of migrating cells is shown in the histogram. Data are expressed as mean ±  SEM (*p <  0.05).

We next investigated contribution of EMT in formation of WT1^+^ cells in the hearts of mutant embryos. To elucidate the network of interactions between EMT regulatory factors, we first assessed the expression of the following transcriptional factors known to promote EMT^17^: *β-catenin, SNAI1 and c-myc,* (a *β-catenin* regulated gene) as well as other genes associated with the mesenchymal state, *Vimentin* (*Vim1*) or the epithelial state, *E-cadherin* in the hearts of mutant and control embryos of day of 16.5. While expressions of epicardial genes-such as *Wt1* and *Tbx18-* were unaffected, the expressions of *β-catenin, Vim1, SNAI1, and c-myc* in PKR1^*Wt1−/−*^ heart were consistently and significantly lower compare to controls (Fig. 5A). Similarly, level of β -catenin protein that is a key player in EMT was reduced in PKR1^*Wt1−/−*^ hearts detected by western blot analyses and demonstrated by the β -catenin immunostaining (Fig. 5B and histogram).

As EMT involves cytoskeletal reorganization, we tested whether gain off function or activation of PKR1 may have any effect on actin cytoskeleton arrangement, thereby causing alteration of cell morphology (e.g., epithelial or mesenchymal-like phenotype). We isolated primary GFP^+^ cells from Wt1creGFP embryonic hearts, overexpressed PKR1 by adenovirus carrying PKR1 cDNA (5MOI)^5^, and analyzed for the distribution of actin fibers by immunofluorescence of phalloidin. Since PKR1 has an intrinsic activity to promote biological activity in the absence of its ligand^7^, PKR1 overexpressing epicardial cells (PKR1-EPDCs) had significant changes in F-actin organization seen as a strong increase in phalloidin fluorescence (mesenchymal phenotype) (Fig. 5C). Consistently, the overexpression of PKR1 in embryonic epicardial cells increased all the EMT markers (except *E-cadherine*) compared to Adv-control infected cells (Fig. 5D). A reduced *E-cadherine* level could be consequences of increased levels of *Twist* and *Snai2* that repress *E-Cadherine* transcript, thus facilitating EMT^18^.

We also examined the EMT events in the epicardial cells of PKR1^*Wt1−/−*^. No F-actin organization was observed in phalloidin stained PKR1 deficient cells upon prokineticin-2 treatment, indicating that PKR1 deficient cells are not able to undergo mesenchymal transition (Fig. 5E left panel). However, the EMT was evident in the control epicardial cells upon prokineticin-2 treatment where the cell adhesion was altered within 1 h as detected by ZO-1, a junctional protein, internalization (5E right panel).

Next we cultured epicardial explants of PKR1^*G5−/−*^ embryos to see whether EMT deficiency exist in this mutant line, by carrying out wound-healing assay. Indeed, the migrating cell number was increased in the control epicardial cells upon prokineticin-2 treatment for 48 h that was abolished by Akt inhibitor LY294002 (Fig. 5F). However, PKR1^*G5−/−*^ mutant cells cannot migrate in response to prokineticin-2. (Fig. 5F lower panel).

**Figure 6:**
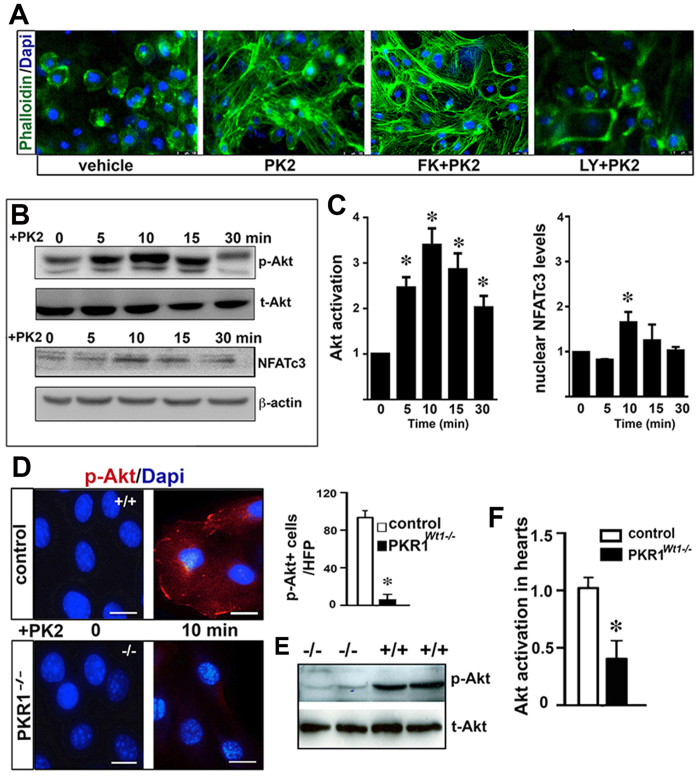
Implication of Akt in prokineticin-mediated EMT. (**A**) Prokineticin-2 (10 nM) induced transformation of the cells from octagonal shape to spindle elongated shape that was blocked by LY294002 (an inhibitor of PI3/Akt kinase) but not NFAT3C inhibitor, FK-506. (**B**) Western blot analyses show Akt activity by phosphorylation and NFATc3 activity by nuclear localization upon PK2 treatment in the indicated time periods. (**C**) Histogram shows quantification of Akt activity and NFATc3 nuclear localization in the epicardial cells upon prokineticin-2 treatment (n =  3). Data are expressed as mean ±  SEM (*p <  0.05). (**D**) Staining of isolated epicardial cells with phosphorylated-Akt antibody revealed no Akt activation in PKR1 deficient cells. (**E**) Western blot analyses of protein extracts derived from hearts with phospho- and total-Akt antibody. Histogram shows activated (p-Akt/t-Akt) levels in hearts (below, n =  3–4 for each group). Data are expressed as mean ±  SEM (*p <  0.05).

Next we investigated involvement of Akt in the PKR1-mediated EMT signaling. Prokineticin-2 treatment of the epicardial cells induced a transition from cuboidal morphology to a spindle-like, elongated shape (Fig. 6A). The phosphoinositide 3-kinase (PI3K)/Akt inhibitor LY294002 (10 μ M) inhibited EMT (Fig. 6A,C histogram). FK-506 (10 μ M) an inhibitor of Nuclear factor of activated T cells (NFATc3), had no effect on prokineticin-2-mediated cellular changes. Consistently, prokineticin-2 did not persistently alter NFATc3 activation by nuclear accumulation (Fig. 6B lower, and C right histogram). Moreover, prokineticin-2 activated Akt by phosphorylating it in Wt1^+^ epicardial cells that was not seen in the PKR1 deficient epicardial cells (Fig. 6D and left histogram). PKR1^*Wt1−/−*^ hearts had also lower levels of phosphorylated Akt compared to control hearts (Fig. 5E,F histogram), establishing the contribution of the PI3K/Akt axis for the PKR1-mediated EMT.

## Discussion

**Figure 7:**
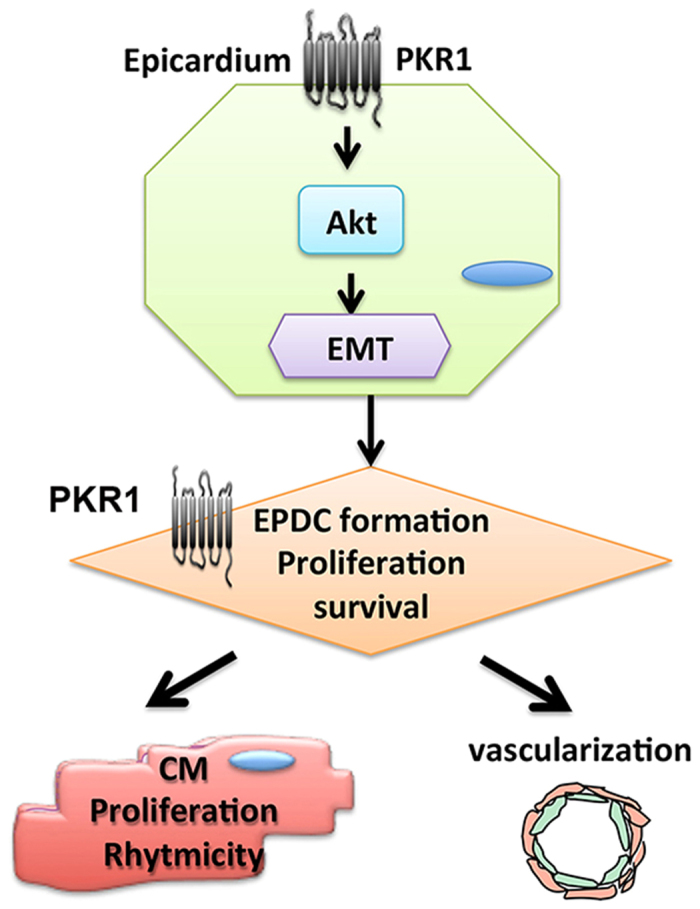
PKR1 signaling during cardiac development. PKR1 signaling via Akt activation in epicardium regulates EMT process that is involved in EPDCs formation. PKR1 in EPDCs regulates proliferation, survival and differentiation that are necessary for growth of ventricular wall, vascularization and cardiomyocytes proliferation, contractility and metabolism.

In this study we provide evidence for the critical role of epicardial-PKR1 signaling in cardiac development, and how defective epicardial signaling manifests in forms of congenital heart diseases in adult heart, focusing on the EMT programming process (Fig. 7).

Using two epicardial- cre lineages, we showed *in vivo* that loss of epicardial PKR1 leads to reduce ventricular expansion and septal defects during embryogenesis and severe hypoplasia and ischemic cardiomyopathy at postnatal stages. Although both mutant hearts had impaired EMT that counts for low EPDCs numbers and their phenotypes, the differences between the two strains (embryonic versus neonatal lethality, hypoplastic ventricle versus hypotrophic atrium, premature formation of z-bands in the subepicardium in the Wt1 cre but not in the Gata5 cre line) can be results of some other as yet unidentified function for this pathway. Alternatively, differences in the timing and expression of their cre^14^ can explain some phenotypical differences between the two strains.

Loss of PKR1 in epicardium of both strains reduces epicardial cell proliferation, migration and adhesion, leading to impaired expansion of subepicardial mesenchyme, suggestive of impaired EMT^19^. EMT is also driven by changes in gene expression and cell morphology. We showed *in vitro* that epicardial cells lose their cell polarity and cell-cell adhesion, and gain migratory properties to become mesenchymal progenitor cells upon prokineticin-2 treatment. However, both PKR1^*G5−/−*^ and PKR1^*Wt1−/−*^ epicardial cells had impaired EMT process in response to prokineticin-2, indicating PKR1 involvement. PKR1 activation or overexpression induced changes in cell morphology, actin cytoskeleton remodeling, and gene expression profile via activating Akt signaling. Activation of the phosphatidylinositol 3-kinase (PI3K) and Akt has been shown to promote EMT in developing heart^20^,^21^. Indeed, inhibition of PI3K/Akt signaling represses epicardial EMT in mice^22^. Similarly, prokineticin-2 dramatically enhances Akt phosphorylation in epicardial cells. Inhibition of Akt phosphorylation blocks the formation of mesenchymal-like cell morphology and migration of the cells. Phosphorylated-Akt positive cell number and protein levels were completely diminished in PKR1 deficient epicardial cells and hearts. In concert, PKR1 signaling has been shown to activate Akt via Gq protein in cardimyocytes to protect these cells against apoptosis^5^. In the endothelial cells PKR1 uses both ERK and Akt signaling pathway to induce proliferation, migration, and angiogenesis^7^. Gq coupled receptors can promote PLC activity and intracellular calcium release. The calcium signaling pathway is involved in activation of calcineurin that dephosphorylates the NFAT transcription factor, triggering NFAT proteins to translocate into the nucleus to control target gene expression^23^. Although NFATc3 was slightly accumulated in the nucleus only 10 min after prokineticin-2 treatment of the epicardial cells, NFAT inhibitor had no effect on EMT events, eliminating the implication of NFAT in regulation of EMT by prokineticin/PKR1 system. All together our data suggest that PKR1 stimulates PI3/Akt for EMT thereby inducing the EPDC formation.

Conditional Rxra^13^, Raldh2^24^ or *β-catenin*^12^ inactivation in epicardial-derived cells utilizing Gata5 cre mice causes epicardial detachment and reduction of the adjacent myocardium compact zone, impairing myocardial growth. This can be due to impaired EMT and the lack of epicardial-derived proliferative signaling^12^. These defects are similar to the ones in our PKR1 mice models. Moreover, prokineticin-2 also promoted proliferation and survival in Wt1^+^ EPDCs in a cell-autonomous manner. Previously, we have shown *in vitro* that prokineticin-2/PKR1 signaling induces adult EPDC proliferation and differentiation into vasculogenic cell type^9^. Impaired vasculogenesis in the epicardial-specific PKR1 mutant mice hearts could be due to impaired EPDC proliferation as well as a defective EPDC differentiation into vasculogenic cell type. Lack of β -catenin in epicardium induces abnormal coronary vessel development^12^,^16^. Downregulation of β -catenin in PKR1^*Wt1−/−*^ epicardium likely accounted for phenotypic similarity of epicardial loss of function of β -catenin and PKR1.

EPDCs have been also shown to provide the cellular substrates for cardiomyocyte proliferation^25^. Our studies uncovered a unique role of epicardium on regulation of cardiomyocyte proliferation, contractile performance, rhythmicity, as well as metabolism. Rhythmicity of PKR1^*G5−/−*^ neonatal cardiomyocytes was lower both at the baseline and upon dobutamine-induced stress condition. Moreover, PKR1^*G5−/−*^ cardiomyocytes had lower calcium handling gene expressions and severe lipid deposition that resulted in impaired contractile performance. The abnormal cardiomyocyte proliferation, contractility and lipid accumulation in mutant hearts could be due to (i) lack of epicardial paracrine factors in mutant hearts, (ii) differentiation of EPDCs into adipocytes^26^. Lack of PKR1 in EPDCs may promote differentiation of EPDCs into adipocytes, as previously shown for preadipocytes that activation of PKR1 suppress adipogenesis^27^. These two phenomena are currently under the investigation in our laboratory.

Due to developmental defects, impaired neonatal heart regenerative capacity and later lipotoxic ischemic cardiomyopathy, adult PKR1^*G5−/−*^ mice exhibited functional impairments and a few of them could survive after experimental myocardial infarction (MI). Previously, we have also shown that transient gene therapy with PKR1 enhances angiogenesis and decreases cardiomyocyte apoptosis after MI^5^. Moreover, PKR1 gene therapy preserves myocardial function by inducing Wt1-positive progenitor cell expansion and migration^4^. In addition, a non-peptide agonist for PKR1 has recently been shown to improve heart function and survival rate after MI. This agonist promotes Wt1^+^ cell proliferation and migration and inhibits cardiomyocyte death^28^. Taken together, these data suggest that PKR1 signaling may influence cardiac remodeling through modulating EPDC activation by EMT and cardiomyocyte homeostasis by paracrine signaling in the developing and injured heart.

PKR1 is expressed in epicardium, cardiomyocytes and endothelial cells in the heart^16^. The total PKR1 knockout mice (PKR1^null^ mice) also show disorders of heart^9^,^10^. However, lack of embryonic lethality in PKR1^null^ mice could be partially due to transient angiogenic gene redundancy in the embryos. For example, perturbation of capillary angiogenesis in the hearts of neonatal PKR1^null^ mice was partially restored at the adult stage via upregulation of hypoxia-inducible factor-1 and proangiogenic factors (e.g. VEGF) due to severe hypoxia. This resulted in reactivation of capillary formation in heart at the expense of the epicardial capillary networks. PKR1^null^ mice had also cardiomyocyte contractile defects and apoptosis partially due to lack of PKR1 signaling in cardiomyocytes (autonomous effect of PKR1). It is clear that PKR1 in the epicardium controls cell autonomous and paracrine signaling in developing hearts. Paracrine factors released from epicardial cells by PKR1 signaling are currently under investigation in our laboratories.

Although myocardially expressed PKR1 induced coronary vessel formation by a paracrine process^9^, here we showed a reciprocal interaction between epicardium and cardiomyocyte via PKR1 signaling. In the other hand, cell autonomous PKR1 signaling in epicardium is indispensable for proper EPDC and myocardial development and function. Finally, our findings are the first to show that epicardial-PKR1 is a key player for EMT process during heart development. Our findings should facilitate development of new strategies for treatment of congenital heart diseases.

## Materials and Methods

The methods were carried out in accordance with the approved guidelines. All the experimental protocols were approved by the Animal Care and Use, and ethics committees of the Bas-Rhin Prefecture (Permit Number: B67-274) with the recommendations in the Guide for the Care and Use of Laboratory Animals of the French Animal Care Committee, with European regulation-approved protocols from Directive 2010/63/EU of the European Parliament on the protection of animals used for scientific purposes.

### Generation of tissue specific PKR1 knockout mice

Mice carrying a *PKR1* gene in which exon 2 are flanked by *loxP* sites^29^ were bred with transgenic mice expressing the Cre recombinase under control of the chicken *Gata5* (G5) promoter-enhancer or Wt1-GFPCre promoter (Jackson Laboratory). The animal experimentation and housing were conducted at the accredited animal experimentation and housing facility of UMR7242 (Register number: C67-218-19).

### Histological and electron microscopy analyses

Male mice or pregnant mice on day 14.5 of gestation were sacrificed by cervical dislocation. Organs and embryos were removed, dissected and frozen for the cutting of frozen sections (5 μ m), which were stained with Mallory tetrachrome^29^.

### Echocardiography and blood pressure measurement

Systolic function in 24 week-old male mice (n =  6 for each group) was assessed by echocardiography in M-mode and two-dimensional measurements as previously described^5^.

### TUNEL and BrdU assays

TUNEL (terminal dUTP nick end-labeling) or BrdU assays were performed with an *in situ* cell death detection kit (Roche), according to the manufacturer’s instructions^9^,^10^.

### Immunostaining

For immunofluorescence assays, frozen tissue or embryo sections were fixed, blocked and incubated with primary antibodies against PECAM-1, Wt1, Dystrophin and β -catenin, Gata5 (Santa Cruz), alpha-SMA (Sigma-Aldrich), PKR1 (IGBMC, Illkirch), GFP (Abcam), Myosin heavy chain epitope (MF20) (DSHB, University of Iowa), Akt (Cell signaling) and active-caspase-3 (Abcam). Phalloidin-488, zonula occluden-1 (ZO-1) (Invitrogen) was used to label F-actin to delineate the cellular cytoskeleton. Antibody binding was detected by incubation with Fluorescein, Alexa 555-, Alexa 488- or Alexa 594-conjugated (Millipore) secondary antibodies or Vectastain ABC peroxidase kit, according to the manufacturer’s instructions.

### Cell isolation

To isolate GFP^+^ cells, pregnant mice were sacrificed by cervical dislocation on day 14.5 of gestation and embryos were dissected and genotyped. Wt1-GFPCre or *PKR1*^Wt1GFP*−/−*^ hearts were dissociated to single cells by digestion with 0.1% collagenase IV (Sigma-Aldrich) and 0.05% trypsin (Invitrogen) in HBSS (Sigma-Aldrich). Then GFP^+^ cells were isolated by FACS sorting as previously described^30^. Glomerular isolation was performed as previously described^31^. Cardiomyocytes were isolated by the Percoll gradient technique as described previously^32^.

### RNA extraction, quantification, and reverse transcription-polymerase chain reaction analysis

Total RNA was isolated from embryos and adult mouse hearts with TRI® Reagent (Molecular Research Center) as previously described^9^.

### Lipid staining and extraction

The cryosectioned frozen neonatal heart samples were stained with Oil Red O (0.5 g in 100 ml isopropanol) for 30 min to visualize the lipid accumulation as previously described. Lipids were extracted from hearts using a modified Bligh and Dyer technique^27^.

### Western Blot assay

Hearts were homogenized in lysis buffer composed of 50 mM Tris-HCl pH 6,8, 1 mM EDTA pH 8.0, 1% NP-40, 1 mM NA_3_VO_4_, 0.1% SDS, 100 mM NaCl, and phosphatase and protease inhibitors. Homogenized samples were centrifuged at 13,000 rpm at 4 °C to obtain protein extracts. The protein concentration was measured by BCA assay (Thermo Scientific) as described in the manufacturer’s instructions.

### Wound-healing migration assay

Confluent epicardial explant cells were wounded with a pipette tip and subjected to prokineticin-2 stimulation for 48 h as previously described^7^.

### Statistical analysis

Data are expressed as means ±  SEM. Multigroup comparisons were carried out by Kruskal–Wallis tests, and Mann–Whitney (for 2 groups). Statistical comparisons for Oil Red O TUNEL, BrdU, Ki67, PECAM-1, Wt1, Calponin and α -SMA etc. staining on heart cryosections were performed using the unpaired Student *t* test and ANOVA. *P* <  0.05 was considered statistically significant for all tests.

## Additional Information

**How to cite this article**: Arora, H. *et al.* Prokineticin receptor-1 signaling promotes Epicardial to Mesenchymal Transition during heart development. *Sci. Rep.* **6**, 25541; doi: 10.1038/srep25541 (2016).

## Supplementary Material

Supplementary Information
