# Supplementary material for: Prokineticin receptor-1 signaling promotes Epicardial to Mesenchymal Transition during heart development
Source: Sci Rep. 2016 May 6;6:25541. doi: 10.1038/srep25541 (PMC4858698; doi:10.1038/srep25541)
Supplement: Supplementary Information [file srep25541-s1.pdf]

Supplemental Material

Prokineticin receptor-1 signaling promotes Epicardial to Mesenchymal Transition during heart development

Himanshu Arora, Mounia Boulberdaa, Rehana Qureshi, Verda Bitirim, Adeline Gasser, Nadia Messaddeq, Pascal Dolle and Canan G. Nebigil

Generation of tissue specific PKR1 knockout mice

Mice carrying a *PKR1* gene in which exon 2 is flanked by *loxP* sites were bred with transgenic mice expressing the Cre recombinase under control of the chicken *Gata5* (G5) promoter-enhancer or Wt1-GFPCre promoter (Jackson Laboratory). All the mice in these experiments had a C57Bl/6 genetic background. Homozygous *PKR1lox/lox* mice were bred with the *G5* promoter-driven Cre transgenic mice to generate *G5-Cre;PKR1lox/WTmice.* These mice were bred with *PKR1lox/lox* mice to generate *G5-Cre;PKR1lox/lox* (i.e., *PKR1G5*$^{-/-}$*)* mice. The same breeding strategy was used to produce mice harboring a Wt1-GFPCre-driven PKR1 gene disruption. The genotype of each mouse was confirmed by PCR. Primers sequences are given in Table 1. The littermates from the same breeding pairs (Cre negative, *PKR1lox/lox)* were used as controls. Animals were housed on a 12-12h light/dark cycle and were fed a standard rodent chow. All experiments were carried out in male mice.

The animal studies were approved by the Animal Care and Use, and ethics committees of the Bas-Rhin Prefecture (Permit Number: B67-274) with the recommendations in the Guide for the Care and Use of Laboratory Animals of the French Animal Care Committee, with European regulation-approved protocols from Directive 2010/63/EU of the European Parliament on the protection of animals used for scientific purposes. The animal experimentation and housing were conducted at the accredited animal experimentation

and housing facility of UMR7242 (Register number: C67-218-19).

Histological and electron microscopy analyses

Male mice or pregnant mice on day 14.5 of gestation were sacrificed by cervical dislocation. Organs and embryos were removed, dissected and frozen for the cutting of frozen sections (5 µm), which were stained with Mallory tetrachrome. For electron microscopy, hearts and embryos were fixed by immersion in glutaraldehyde, postfixed by incubation in osmium tetroxide and embedded in epoxy resin, using standard methods.

Echocardiography and blood pressure measurement

Systolic function in 24 week-old male mice (n=6 for each group) was assessed by echocardiography in M-mode and two-dimensional measurements. Mice were sedated with an induction dose of 3% isoflurane gas 1 minute and a maintenance dose of 1.25 to 1.50% isoflurane gas in oxygen. Male *PKR1*$^{G5-/-}$ mice at 12 weeks of age (weight 20-25 g) were used to produce myocardial infarction (MI) by ligation of the left anterior descending coronary artery as described [5].

TUNEL and BrdU assays

TUNEL (terminal dUTP nick end-labeling) assays were performed on the cryosectioned hearts or serum-starved epicardial cells for (48h) with an *in situ* cell death detection kit (Roche), according to the manufacturer's instructions. For BrdU staining, mice received 100 µL (intraperitoneally) of BrdU (20 µg/mL) 4 h before the hearts were collected for the immunostaining experiments.

Immunostaining

For immunofluorescence assays, frozen tissue or embryo sections were fixed, blocked and incubated with primary antibodies against PECAM-1, Wt1, Dystrophin and β-catenin, Gata5 (Santa Cruz), alpha-SMA (Sigma), PKR1 (IGBMC, Illkirch), GFP (Abcam), Myosin

heavy chain epitope (MF20) (DSHB, University of Iowa) and active-caspase-3 (Abcam). Phalloidin-488 (Invitrogen) was used to label F-actin to delineate the cellular cytoskeleton. Phosphor- Akt (Cell Signaling) was used to detect activated Akt positive cells upon prokineticin-2 treatment at the indicated times. Cryosectioned hearts or cells were stained with Ki67 antibody to detect the proliferating cells. Cells were incubated with prokineticin-2 or FGF for 48h. Then cells were fixed and stained with Ki67 antibody. Antibody binding was detected by incubation with Fluorescein, Alexa 555-, Alexa 488- or Alexa 594-conjugated (Millipore) secondary antibodies and then Vectastain or ABC peroxidase kit, according to the manufacturer's instructions. Data were analyzed using a Leica fluorescence microscope. For quantification of cell numbers, comparable areas were randomly chosen from each group and numbers of positive cells were counted. At least 6 areas were scored for each sample.

Cell isolation

To isolate GFP+ cells, pregnant mice were sacrificed by cervical dislocation on day 14.5 of gestation and embryos were dissected and genotyped. Wt1-GFPCre or *PKR1*$^{Wt1GFP-/-}$ hearts were dissociated to single cells by digestion with 0.1% collagenase IV (Sigma-Aldrich) and 0.05% trypsin (Invitrogen) in HBSS (Sigma- Aldrich). Then GFP+ cells were isolated by FACS sorting, as previously described. Sorted cells were collected and cultured into DMEM with 15% FBS on 2% gelatin (epicardial) or 1% collagen (glomerular cells) coated dishes. Glomerular isolation was performed as previously described. Cardiomyocytes were isolated by the Percoll gradient technique as described previously. In the other settings beating rate in response to dobutamine was determined by time laps recording of isolated cardiomyocytes utilizing an inverted Leica microscope.

Cell infection and treatments: Prior to infection, cells were washed with sterile PBS and then incubated with serum free medium containing adenovirus (Adv-control or Adv-PKR1, 1×10^{7}pfu/ml, 5 M0I) overnight at 37°C. Virus infected primary epicardial cells were

incubated with recombinant human prokineticin-2 (final concentration 10nM, Peprotech) for 48 h to stimulate EMT that was detected by Phalloidin-488 staining. In the other settings the infected cells were incubated 1h with PI3/Akt inhibitor LY29400 (Promega), (10μM) or NFAT inhibitor FK-506 (Sigma-Aldrich) (10μM) before incubating the cells with prokineticin-2 (10nM) for 48h.

RNA extraction, quantification, and reverse transcription-polymerase chain reaction analysis

Total RNA was isolated from embryos and adult mouse hearts with TRI®Reagent (Molecular Research Center) as previously described or RNeasy columns (Qiagen). RNA (1 ug) was reverse transcribed into cDNA using the First Stand Synthesis Kit (Qiagen, Germantown, MD, USA) and then subjected to real-time qPCR analysis of genes. Data in the form of threshold cycle numbers (Ct) were analyzed with Bio-Rad qPCR software version 2.0. Primer sequences are shown in Table I. Fold changes in gene expression for pairwise comparison was calculated using the ΔΔCT method.

Lipid staining and extraction

The cryosectioned frozen neonatal heart samples were stained with Oil Red O (0.5 g in 100 ml isopropanol) for 30 min to visualize the lipid accumulation as previously described. Images were captured at a magnification of 20× or 40× with a digital microscope camera system (Leica). ImageJ software (National Institutes of Health) was used to convert bright field (24 bit) images of Oil red O stainings to 8 bit images. Lipids were extracted from hearts using a modified Bligh and Dyer technique. In brief, tissue was homogenized in an ice-cold chloroform/methanol/water (2:1:0.8) solution. Additional chloroform and water was added to separate layers, and the mixture was centrifuged at 12,000 *g.* Following centrifugation, the chloroform layer was extracted and evaporated. The extracted dry lipids were quantified as weight and normalized by tissue weight.

Western Blot assay

Hearts were homogenized in lysis buffer composed of 50 mM Tris-HCl pH 6,8, 1 mM EDTA pH 8.0, 1% NP-40, 1 mM NA_3VO_4, 0.1% SDS, 100 mM NaCl, and phosphatase and protease inhibitors. Homogenized samples were centrifuged at 13,000 rpm at 4°C to obtain protein extracts. The protein concentration was measured by BCA assay (Thermo Scientific) as described in the manufacturer's instructions. Approximately 20-40 μg of protein for each sample were separated by 12% SDS/PAGE, transferred to a PVDF membrane, and incubated with beta catenin (Abcam), and GAPDH (Santa Cruz) at 4°C overnight.

Isolated epicardial cells were plated and grown for 24h. Next, the cells were washed and cultured for 12 h in the above-mentioned serum-free medium, supplemented with only 1% fetal calf serum. The cells were then incubated with either prokineticin-2(10nM) or vehicle alone for 0, 5, 10, 15, or 30 min and harvested with a lysis buffer (50 mM Tris-HCl pH 7.0, 1 mM EDTA, 100mM NaCl, 0.1% SDS, 1% NP-40, 1 mM Na3VO4, 1 mg/mL aprotinin, 1 mg/mL pepstatin, and 1 mg/mL leupeptin). The whole-cell lysates were centrifuged at 12 000 × g for 15 min at 4°C. The cell debris was removed. Cytoplasmic fractions from cultured cells were prepared using Subcellular Protein Fractionation Kit for Cultured Cells (Thermo Scientific) and nuclear isolation kit, employing the nuclear protein extraction buffer (20 mM Tris–HCl, pH 7.6, 50 mM KCl, 400 mM NaCl, 1 mM EDTA, 0.2 mM PMSF, 5 mM β-mercaptoethanol, aprotinin (1000 U/ml), 1% Triton X-100, and 20% glycerol. 30μg of total protein, 10μg of cytosolic, or nuclear protein were used for Western blot analyses. For Akt activity, anti-phospho-Akt antibody Ser (473) (Cell Signaling) was used. After three washes with PBS-T, the membrane was incubated for 1 h at room temperature with gentle shaking with a horseradish peroxidase-conjugated corresponding anti-IgG antibody in PBS-T containing 0.5% of the fat-free milk powder. The expected bands were visualized after 5-min incubation to induce enzyme-linked chemiluminescence (Amersham Pharmacia), and then the blots were washed, stripped, and reprobed with an Total -Akt

antibody (Cell Signaling) or vinculine (Cell Signaling) or actin (Santa Cruz) as internal control, followed by incubation with a suitable secondary antibody. The intensity of the resulting bands for phospho-Akt, beta catenin signals were quantified by Image J software and normalize to total amounts of the corresponding total-Akt or actin or vinculine protein, respectively.

Wound-healing migration assay

Epicardial explant cells were counted at seeding, and equal numbers of cells (10^5 cells per well) were incubated in a six-well plate containing DMEM supplemented with 10% FCS for 48 h. Confluent cells were kept in DMEM supplemented with 2% FCS for overnight and were then wounded with a pipette tip and subjected to prokineticin-2 stimulation. The cells were cultured for 48 h and then photographed again. To distinguish between enhanced restitution by migration and proliferation, cells were treated by 0.5 μm/ml mitomycin C for 2 h before wounding assays were performed.

FIGURES

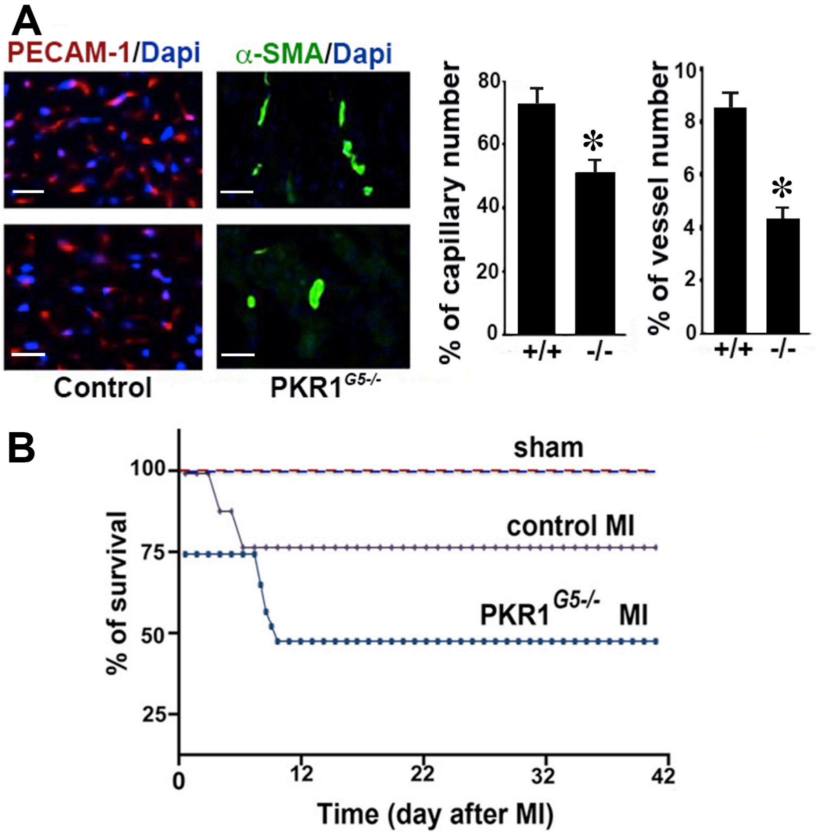

Figure S1. Number of capillaries and coronary arteries in mutant hearts and survival rate of mice after MI.

(A) PECAM-1-positive capillary numbers and α-SMA-positive capillary arteries were reduced in $PKR1^{G5-/-}$ mutant adult hearts. 10 high-power microscopic fields (x 40) from 5-6 sections per organ were analyzed (5-6 mice per genotype). Data are expressed as mean±SEM. *p<0.05. **(B)** The survival rate of the mutant mice was dramatically reduced after coronary ligation, *i.e.* in a mouse model of myocardial infarction (n=8-10). Note that during the MI process 25% mutant mice has been died.

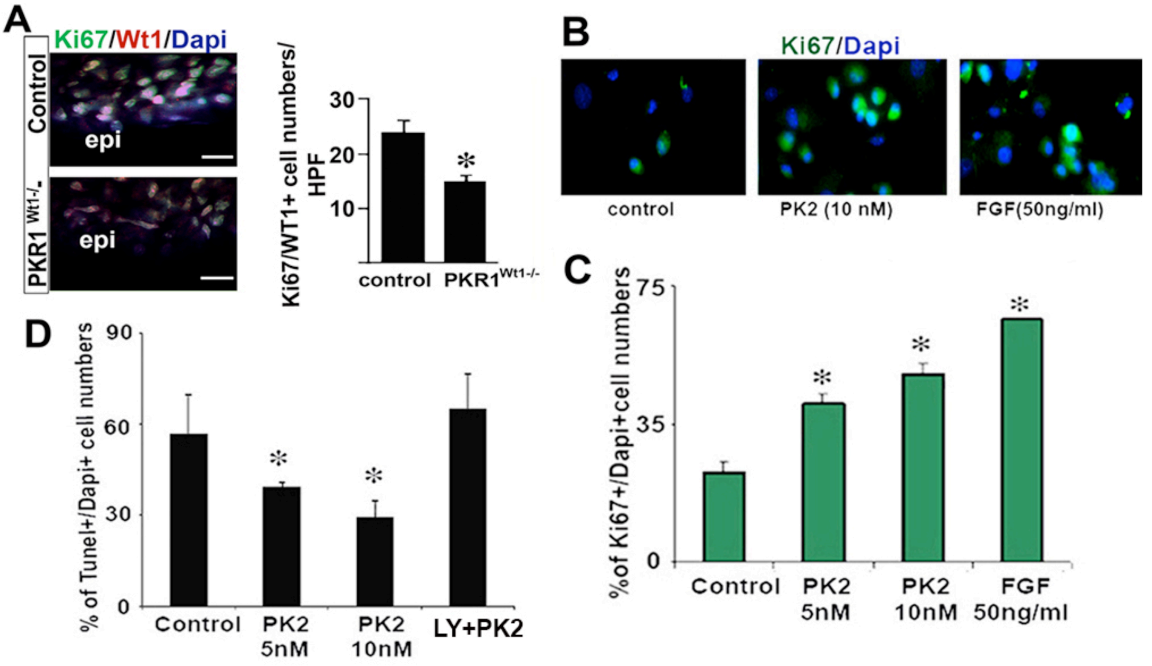

Figure S2. Effects of Prokineticin-2 on survival and proliferation of EPDCs. (A) Co-immunostaining of cryosectioned hearts with Ki67 and Wt1 antibodies. Histograms show that Ki67+/Wt1+ cell numbers were reduced in PKR1$^{Wt1-/-}$ hearts. 10 high-power microscopic fields (x 40) from 5-6 sections per organ were analyzed (5-6 mice per genotype)**. (B) and (C)** PK2 promotes EPDCs proliferation in dose dependent manner that was comparable with a known EPDC mitogen, FGF. **(D)** PK2 protects EPDCs against serum deprivation-mediated apoptosis detected by Tunel in dose-dependent manner. Cytoprotective effects of PK2 were inhibited by PI3K/Akt inhibitor (LY294002,10μM). Data are expressed as mean±SEM. *p<0.05 (n=3-4).

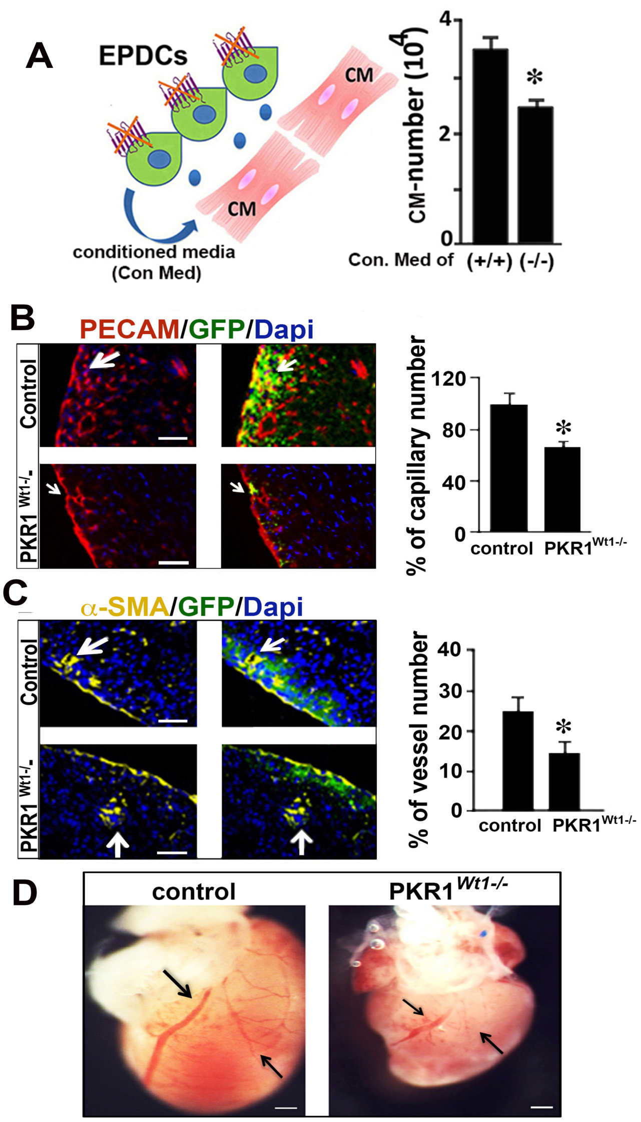

Figure S3. PKR1$^{Wt1-/-}$mice exhibit vascularization defects in epicardium. (A) Illustration of cardiomyocyte (CM) culture in the conditioned media of control or mutant EPDCs. Condition medium was collected 24h after culturing EPDCs. Proliferation rate of cardiomyocytes were reduced when they cultured in condition medium derived from mutant epicardial cells. Data are expressed as mean±SEM. *p<0.05. **(B)** Co-immunostaining of cryosectioned hearts with GFP and PECAM-1, or **(C)** GFP and α-SMA antibodies. Histograms show that PECAM-1^{+} cells (indicative of capillary formation) and α-SMA^{+} vessel numbers were reduced in PKR1$^{Wt1-/-}$ hearts. 10 high-power microscopic fields (x 40) from 5-6 sections per organ were analyzed (5-6 mice per genotype). Data are expressed as mean±SEM. *p<0.05. **(D)** Evans blue staining of hearts visualizes the

coronary artery defects in PKR1$^{Wt1-/-}$ hearts.

Tables

Table 1. Primers

Genes	Forward primer (5' → 3')	Reverse primer (5' → 3')
PKR1 (floxed)	GACTGGACATCTAGTGGTAGTCAGG	GGGTGTGAGGTGGGATTAAGTCAC
Cre	ATTCTCGTGGAACTGGATGG	GGACAGGTAATGGTTGTCTGG
Col1a1	GCCAAGAAGACATCCCTGAAG	TGTGGCAGATACAGATCAAGC
Col1a2	CACCCCAGCGAAGAACTCATA	GCCACCATTGATAGTCTCTCC
Col3a1	TTTGTGCAAAGTGGAACCTG	TGGACTGCTGTGCCAAAATA
Gata4	TCTCACTATGGGCACAGCAG	GCGATGTCTGAGTGACAGGA
ANF	CCTGTGTACAGTGCGGTGTC	CCTGCTTCCTCAGTCTGCTC
α MHC	GAGATTTCTCCAACCCAG	TCTGACTTTCGGAGGTACT
β MHC	CTACAGGCCTGGGCTTACCT	TCTCCTTCTCAGACTTCCGC
β actin	CATCTTGGCCTCACTGTCCA	GGGCCGGACTCATCGTACT
Gapdh	TGAGGCCGGTGCTGAGTATGTCG	CCACAGTCTTCTGGGTGGCAGTG
P.lamban	TCT CCC TAC TTT TGC CTT CCT G	TCT CAC AAA GCT GTT CTC AGC
RyR	GAA TCA GTG AGT TAC TGG GCA	CTG GTC TGA GTT CTC CAA AAG
Serca2a	CAA TAC TGG AGT AAC CGC	AAC TTC TCT GGA GAG GC
β-Catenin	ATGGAGCCGGACAGAAAAGC	CTTGCCACTCAGGGAAGGA
Vim1	CGGCTGCGAGAGAAATTGC	CCACTTTCCGTTCAAGGTCAAG
Snai1	CACACGCTGCCTTGTGTCT	GGTCAGCAAAAGCACGGTT
MYC	ATGCCCCTCAACGTGAACTTC	CGCAACATAGGATGGAGAGCA
STEAP1	GGTCGCCATTACCCTCTTGG	GGTATGAGAGACTGTAAACAGCG
HSP90AB1	GTCCGCCGTGTGTTCATCAT	GCACTTCTTGACGATGTTCTTGC
GSK3B	TGGCAGCAAGGTAACCACAG	CGGTTCTTAAATCGCTTGTCCTG
TWIST1	GGACAAGCTGAGCAAGATTCA	CGGAGAAGGCGTAGCTGAG
N-cadh	CTCCAACGGGCATCTTCATTAT	CAAGTGAAACCGGGCTATCAG
E-cadh	TCGGAAGACTCCCGATTCAAA	CGGACGAGGAAACTGGTCTC

Table 2. Echocardiographic analyses (n=6 each, 24 weeks old, around 25g)

**Echocardiographic data**		
	Control	PKR1$^{G5-/-}$
Heart rate (beats/min)	484.8±26.7	486±22.35
LV mass (mg)	165.32±33.51	106.48±4.72*
**Diastolic parameters**		
LVID (mm)	3.37±0.24	3.73±0.12
LVPW (mm)	1.27±0.14	1.02±0.12 *
LV vol (µl)	56.92±10.72	59.75±4.14
**Systolic parameters**		
LVID (mm)	1.76±0.18	2.46±0.28*
LVPW (mm)	1.87±0.07	1.45±0.15*
LV vol (µl)	10.95±2.22	23.39±5.64*
Ejection fraction (%)	80.04±2.77	62.83±7.27*
Fractionnal shortening (%)	48.09±3	34.76±5.93*
E wave (mm/s)	1246.94±129.95	1131.31±214.97
A wave (mm/s)	890.99±98.17	746.62±179.90
EA ratio	1.42±0.08	1.69±0.41
IVRT (ms)	14.35±2.37	11.67±3.43
IVCT (ms)	9.85±1.48	13.25±1.87*
